# Factors associated with increased risk of playing-related disorders among classical music students within the Risk of Music Students (RISMUS) longitudinal study

**DOI:** 10.1038/s41598-023-49965-7

**Published:** 2023-12-22

**Authors:** Cinzia Cruder, Emiliano Soldini, Nigel Gleeson, Marco Barbero

**Affiliations:** 1 Rehabilitation Research Laboratory 2rLab, Department of Business Economics, Health and Social Care, University of Applied Sciences and Arts of Southern Switzerland, Manno, Switzerland; 2 Centre for Health, Activity and Rehabilitation Research, Queen Margaret University, Edinburgh, UK; 3 Competence Centre for Healthcare Practices and Policies, Department of Business Economics, Health and Social Care, University of Applied Sciences and Arts of Southern Switzerland, Manno, Switzerland

**Keywords:** Health care, Occupational health

## Abstract

Playing-related musculoskeletal disorders (PRMDs) are common among musicians but longitudinal data on risk factors are limited. The aim of the study was to longitudinally identify the factors associated with increased risk of PRMD onset among music students enrolled in different pan-European music institutions. A further goal was to assess the occurrence of PRMDs during a 12-month period. A total of 442 students without PRMDs from 56 European music universities completed a web-based questionnaire on lifestyle and practice habits, health history, physical activity, psychological distress, perfectionism, and fatigue. PRMD onset was assessed prospectively at 6 and 12 months. Logistic regression analysis showed that changes in physical activity level (6-month *AOR* = 2.343, 12-month *AOR* = 2.346), increased levels of fatigue (6-month *AOR* = 1.084, 12-month *AOR* = 1.081) and socially-prescribed perfectionism (6-month *AOR* = 1.102) were significantly associated with PRMD onset, which had occurred in 49% of participants during 12 months. Musculoskeletal complaints reported at baseline (6-month *AOR* = 0.145, 12-month *AOR* = 0.441) and changes to BMI (12-month *AOR* = 0.663) limited the onset of PRMDs. The study’s novel longitudinal findings were appraised critically within the contexts of potential factors for PRMD onset and evidence-based preventive strategies to minimise the impact of PRMDs.

## Introduction

Due to a highly competitive environment^1–3^, many musicians might suffer from playing-related musculoskeletal disorders (PRMDs), interfering with their ability to play^4^ and thus posing significant barriers to performing effectively^4–7^. PRMDs might have significant effects on musical practice, performance and sometimes even on musicians’ career-pathways, being present already at the very beginning of tertiary education^8–22^. As current music students are going to be future teachers and professors, preventive strategies should be considered fundamental in order to adjust musicians’ attitudes toward health^23^. Spreading a culture of health promotion and prevention of PRMDs could be the key to safeguarding the health of future professionals, while simultaneously countering prevalence of disorders amongst music students.

Ideally, preventive strategies for PRMDs amongst music students require longitudinal exploration of factors contributing most to PRMDs’ development. Several risk factors have been explored in the literature and growing evidence has shown a relationship between musculoskeletal complaints and being a female playing a stringed instrument^24^, psychological stressors such as performance anxiety, depression and stress^5,6,24–26^. In addition, a recent study describing self-reported causes of musicians’ musculoskeletal symptoms collected using a musician-driven approach (i.e., participants were free to list their perceived risk factor) revealed that position while playing, poor technique and duration of musical activity, lack of physical activity, stress and poor sleep were the most frequently reported risk factors^27^. Furthermore, amongst other studies that include perceived factors attributed by the musicians themselves as being the causes of their condition, poor practice habits (e.g., heavy playing loads, insufficient rest breaks)^9,12,27–29^ along with excess perceived muscle tension and muscle fatigue^9,29,30^ were frequently reported.

Nevertheless, the contemporary literature offers only limited appraisals of possible relationships amongst musicians’ activities and the development of PRMDs due to the presence of methodological concerns (e.g., small sample sizes)^31^, heterogeneity among studies especially regarding terminology^31,32^ and outcome measures^33^, as well as the lack of longitudinal studies^31^. Ballenberger and colleagues^34^ developed a novel prospective and comparative cohort study describing musculoskeletal health complaints and associated risk factors among students of music and other disciplines. Monthly complaints in music students were predicted by an ongoing complaint and by reduced physical functioning^35^. Further longitudinal studies are needed to corroborate these findings and to offer reliable and consistent results, monitoring students over their entire training.

Moreover, in order to understand the aetiology of musculoskeletal complaints among musicians, it is important to disentangle PRMDs effectively from general musculoskeletal complaints in order to precisely describe complexities of risk for developing PRMDs and to moderate their effects^14^. Using a well-defined criterion for PRMDs such as that defined and operationalised by Zaza et al.^7^ within the latter process would be expected to reduce bias when reporting results, identifying more precisely factors for the specific occurrence of PRMDs through minimised heterogeneity within results^31–33^ and speculatively, may enhance preventative strategies for the treatment of PRMDs, especially if candidate factors were investigated longitudinally.

Accordingly, the Risk of Music Students (RISMUS) research project, a 12-months longitudinal multicentre investigation, described the influence of specific features (e.g., factors reflecting demographics, health-related status and the playing of a musical instrument) in the development of PRMDs among music students at different stages of their training (i.e., from secondary to tertiary education). The main aim of this paper was to report the factors associated with the onset of PRMDs within 6 and 12 months from the student's baseline assessment^14^. A secondary aim was to assess the occurrence of PRMDs during a 12-month period.

## Methods

This study focuses on the longitudinal results of the RISMUS research project, whose rationale and protocol of investigation has been described previously^14,36^. RISMUS has been conducted between November 2018 and January 2020 to obtain self-reported data from a large population of music students enrolled in different academies and music universities in Europe at baseline, and then at 6- and 12-months follow-up to monitor and characterise the PRMD development at different stages of musical training. The research project was granted ethical approval by the Research Ethics Committee of Queen Margaret University of Edinburgh (REP 0177). The authors confirm that all methods were performed in accordance with relevant guidelines and regulations.

### Participants

The student registries of 56 conservatories and universities of music in Europe, which have been described in a previous work^14^, circulated the recruitment e-mail with the link to a web-based questionnaire to their student groups between November 2018 and January 2019. Informed electronic written consent was obtained from all participants prior to data collection.

Eligibility for inclusion within the study’s whole sample (n = 850) required participants to have reported being free from any PRMDs at baseline of the RISMUS project, to have been at least 18 years old, have provided written consent to participate in the study and to have been secondary- and/or tertiary-level students playing a musical instrument commonly used in classical music as a main subject. Participants were excluded if within the 12 months prior to baseline, they self-reported PRMDs, or an episode of severe and highly disabling neurological and/or rheumatic condition (e.g., fibromyalgia syndrome, rheumatoid arthritis, focal dystonia), a psychological condition (e.g., diagnosed severe borderline personality disorders), accidents (e.g., motor vehicle accident) and/or surgery of the upper limbs or spine^14–16,36^. Exclusion criteria were verified at each assessment.

### Procedure

**Figure 1:**
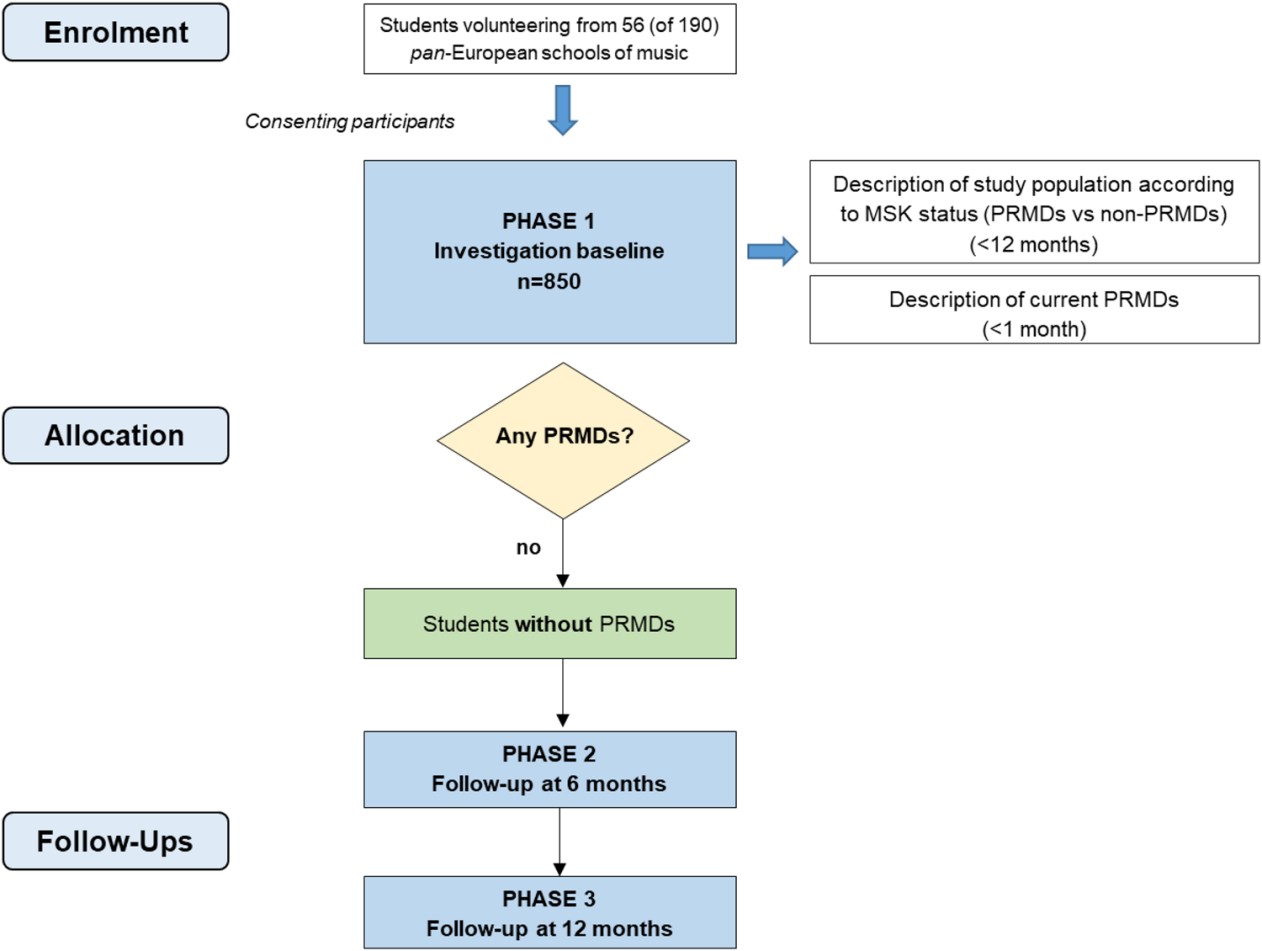
Research planning chart including main features of the cross-sectional studies and longitudinal study. MSK, Musculoskeletal; PRMD, Playing-related musculoskeletal disorder.

The research study consisted of a baseline assessment, a first follow-up at 6 months and a second follow-up at 12 months (see Fig. 1). After the baseline (i.e., Phase 1) participants were coded according to their PRMD status in the past 12 months as a variable to be used amongst the whole sample. In order to identify the factors associated with the onset of PRMDs among participants and describe the one-year occurrence, the cohort of music students without PRMDs at baseline was followed and reassessed at 6 and 12 months.

### Materials

The web-based questionnaire included different self-reported research measures, which are divided in the main outcome (i.e., PRMDs) and measures deemed to be potential risk factors for the development of PRMDs. A detailed description of the research measures is available in the published protocol^36^ and in a previous work^14^.

The questionnaire started with a general section containing questions about personal background and lifestyle (i.e., age, gender, self-reported weight and height, nationality, smoking status and sleeping habits), practice habits (i.e., instrument, number of hours of practice, years of experience; warm-up and preparatory exercises, breaks during practice) and health history (i.e., any painful MSK complaint and current medication, as well as major past injuries/accident and conditions according to the excluded criteria). Subsequently, the single question (‘Do you have pain, weakness, lack of control, numbness, tingling or other symptoms that interfere with your ability to play your instrument at the level you are accustomed to?’) was asked of participants in accordance with Zaza et al.^7^, to identify the presence of PRMDs. Finally, the questionnaire included the following validated research measures:

*Self-rated health*^37^ for the assessment of health status. This measure has been shown to have good test–retest reliability (overall agreement rates between 85 and 90% and kappa values between 0.63 and 0.72)^38,39^.

*International Physical Activity Questionnaire-short form* (IPAQ-SF)^40–42^ for the assessment of physical activity participation levels. The IPAQ-SF showed adequate concurrent validity when compared to the long form (*rho* = 0.67; 95% CI 0.64 to 0.70) and alternative short research measures (*rho* = 0.58; 95% CI 0.51 to 0.64) and criterion validity, when compared to accelerometers (*rho* = 0.30; 95% CI 0.23 to 0.36)^43^. Furthermore, its repeatability exhibited an acceptable level (*r* > 0.69) for people of different nationalities^43^.

*Kessler Psychological Distress Scale* (K10)^44^ for the assessment of psychological distress. This scale has shown moderate reliability (kappa and weighted kappa scores range between 0.42 to 0.74) amongst the general population in the USA^44^, a good construct validity and reliability (Cronbach’s alpha of 0.84 and omega total of 0.88)^45^ and a high level of internal consistency, with a Cronbach’s alpha of 0.88 and an ordinal alpha of 0.92^46^.

*Multidimensional Perfectionism Scale-short form* (HFMPS-SF)^47,48^ for the assessment of perfectionism. Test–retest results for reliability have been adequate with significant correlations (*p* < 0.05) over two points in time for each sub-scale of perfectionism (i.e., self-oriented, other-oriented, and socially prescribed perfectionism)^47,49^.

*Chalder Fatigue Scale* (CFQ 11)^50^ for the assessment of fatigue. The scale has been shown to have good internal consistency^50^, with Cronbach alpha ranging between 0.86 and 0.92^50–53^.

As mentioned in previous works^14–16^, participants were allocated into six categories according to the classification of Kok et al.^54^ that focused on elevation of the arm while playing (i.e., ≥ 40° abduction and/or ≥ 40° forward flexion) and which were derived from work by Nyman et al.^55^.

### Statistical analysis

Descriptive statistics were used to present the variables included in the analysis. Categorical variables were described through frequency distributions, while continuous variables were described using synthetic indicators (median and interquartile ranges [IQR]).

PRMD onset at the 1st and 2nd follow-ups (i.e., temporal horizon of 6 months and 12 months, respectively) was measured in both cases as a binary variable (i.e., yes, no), along the lines of the previous baseline study^14^. In this instance, however, participants reporting no history of MSK complaints and those reporting a MSK complaint that was not interfering with their ability to play (i.e., they reply ‘no’ to Zaza et al.’s question at either 6-month or 12-months assessments) were merged into a single category. For time-invariant variables or variables with a known variation over time (e.g., age), only the baseline was considered. For time-variant variables, the analysis considered both the baseline and the evolution during the follow-up period. For time-variant variables of the first follow-up, the evolution was considered as the difference between the first follow-up and the baseline. For those of the second follow-up, the difference between the first follow-up and the baseline was considered for students with a PRMD onset within 6 months, while the difference with the second follow-up was retained for students who reported the onset of a PRMD after 6 months. Additionally, bivariate analysis explored the associations between the onset of PRMD within 6 and 12 months, and the independent variables (i.e., demographic variables, those with reference to the health-related status and those related to the playing of musical instruments). The chi-square test was used to analyse the association between PRMD onset and categorical variables, while the associations with continuous variables were assessed through the Mann–Whitney U tests. Subsequently, according to the binary nature of the outcome variable (0 = not PRMD onset; 1 = PRMD onset), two logistic regression models were estimated with the aim of identifying variables associated with the dependent variable PRMD onset at a multivariable level. Each model was estimated twice corresponding to forward selection and backwards elimination of variables, respectively, for data available at each of the 6- and 12-month assessments, involving a stepwise approach and the same set of candidate independent variables.

In accordance with the baseline data analysis^14^, bivariate and multivariable analyses were conducted on the responses of the overall sample and on those of a sub-sample of participants not taking any supplements, contraceptives and/or medications to examine whether these treatments could have prejudiced or affected the responses. These sensitivity analyses were considered important because medications such as pain killers or antidepressants might have interfered with the perception of pain and MSK complaints and therefore affecting the interpretation of the results.

## Results

**Figure 2:**
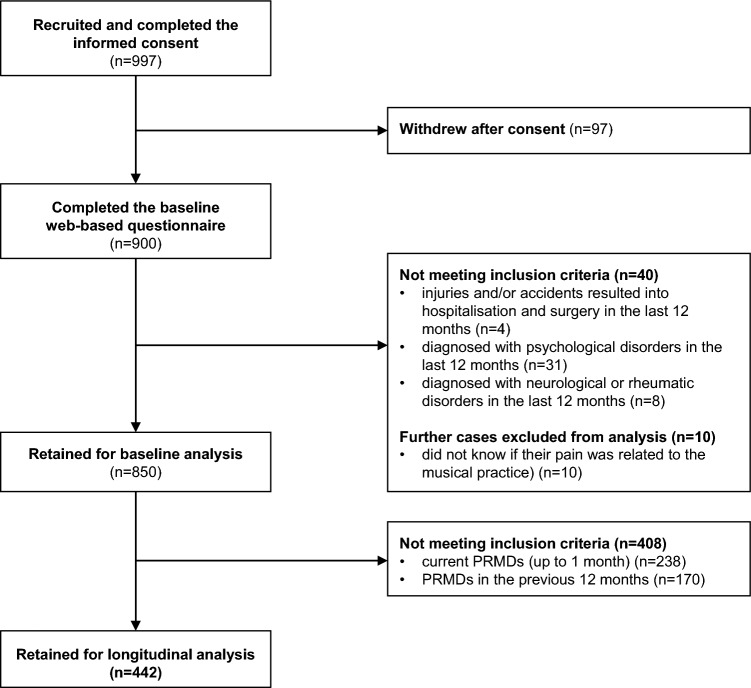
Flowchart of participant selection for the longitudinal analysis. PRMD, playing-related musculoskeletal disorder.

Of the 850 students assessed at baseline, a total of 442 participants (58.1% women; median age 22 years, range 18–48) did not report the presence of PRMDs and were therefore retained for the analysis (see Fig. 2). Specifically, 290 (65.6%) self-reported having no MSK complaints and 152 (34.4%) considered their MSK complaint not interfering with their ability to play.

**Figure 3:**
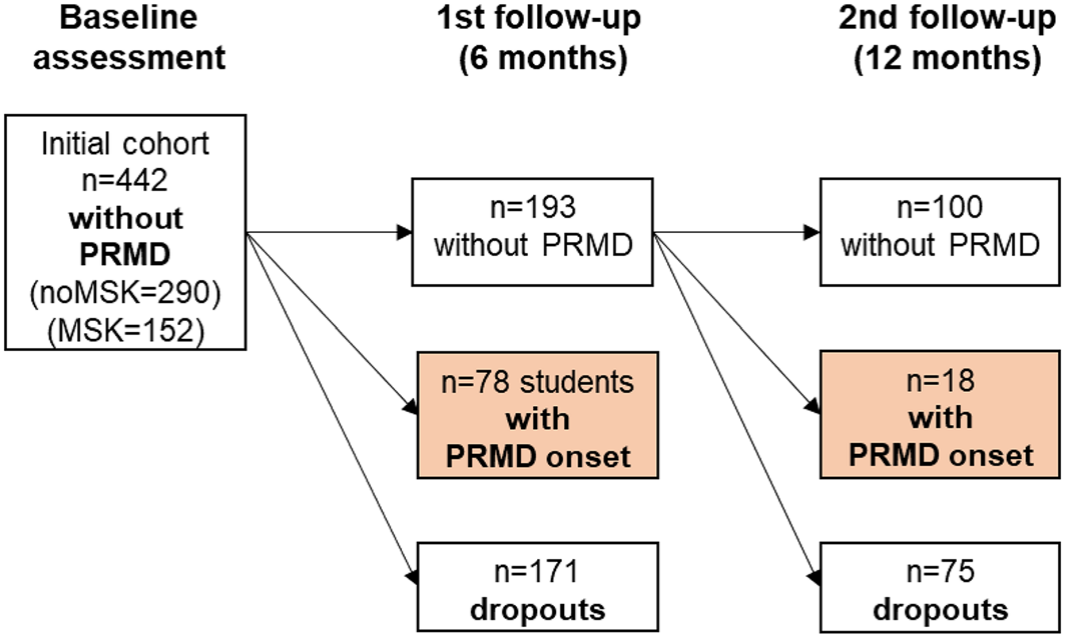
Students’ cohort disposition. PRMD, playing-related musculoskeletal disorder.

A total of 271 participants (78 with PRMDs and 193 without PRMDs) were available for the 6-month analysis as there had been 171 participants dropping-out. Additionally, the data of the 78 participants who developed a PRMD within 6 months was combined with those of the 118 participants that completed both follow-ups, obtaining a total of 196 cases available for the 12-month analysis (see Fig. 3).

**Figure 4:**
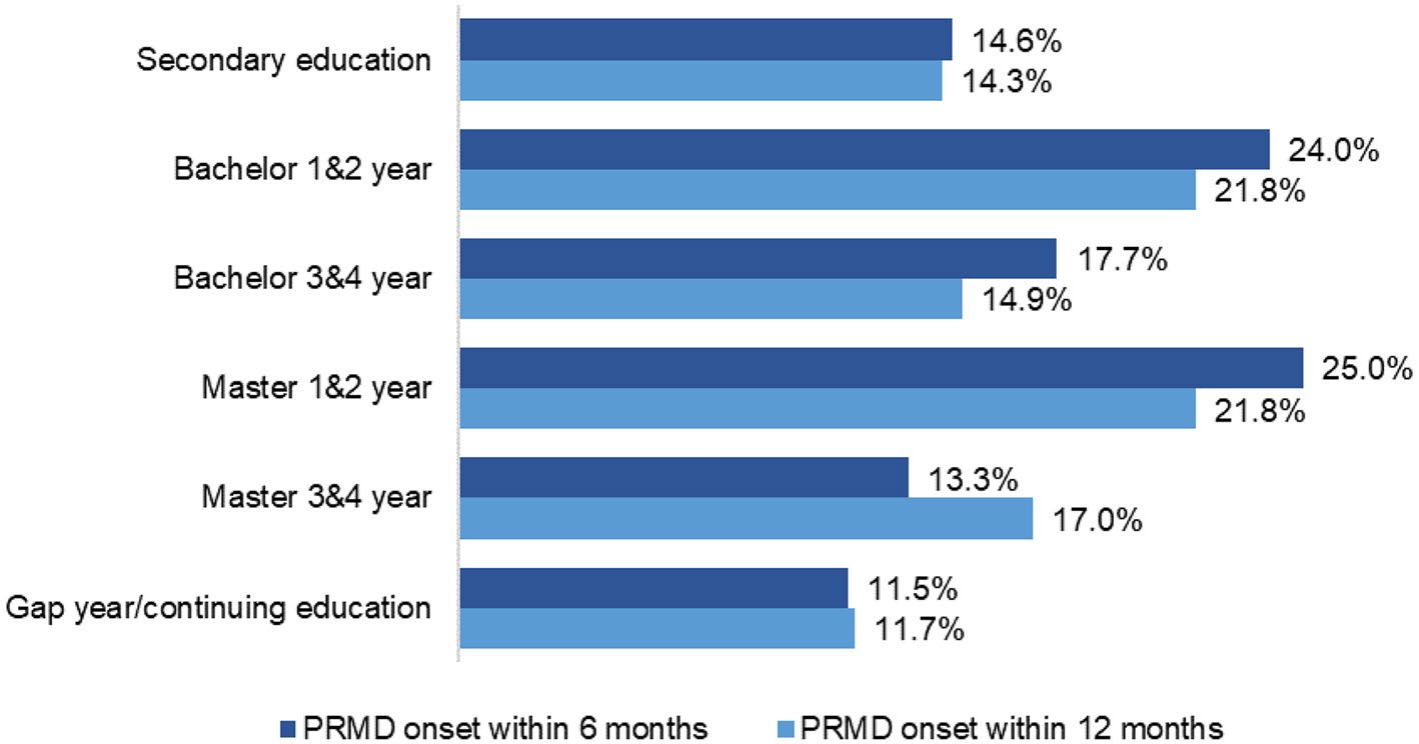
Occurrence of PRMDs according to the academic level. PRMD, playing-related musculoskeletal disorder.

A total of 96 students out of 196 (49%) developed a PRMD within 12 months (see Table 1). When considering the single occurrences according to the academic level (see Fig. 4), first- or second-year Bachelor students developed the highest rate of PRMDs (24.0%) within 6 months, whereas at 12 months, their figure was the same as that of first- or second-year Masters students (21.8%). In comparison with the latter groups, PRMD occurrence was lower in the Bachelor 3&4-year group (17.7% within 6 months and 14.9% within 12 months), the secondary education group (14.6% within 6 months and 14.3% within 12 months) and Masters 3&4-year group (13.3% within 6 months and 17.0% within 12 months). Lastly, the lowest occurrence was reported by the Gap year and continuing education group, with 11.5% within 6 months and 11.7% within 12 months.

**Table 1:** PRMD onset within 6 months (1st follow-up) and 12 months (2nd follow-up).

Variable		Within 6 months (n = 271)		Within 12 months (n = 196)	
		n	%	n	%
PRMD onset	No	193	71.2%	100	51.0%
	Yes	78	28.8%	96	49.0%

PRMD, playing-related musculoskeletal disorder.

Descriptive statistics can be found as Supplementary Table S1 and Supplementary Table S2, while the results of bivariate analysis can be found as Supplementary Table S3.

The results of the logistic regression analysis are presented in Table 2, which reports the Adjusted Odds Ratios (AOR) for the independent variables, along with their standard deviations, the number of observations upon which the models were based, the McFadden Pseudo-R^2^ and the area under the ROC curve. The latter yielded 0.72 for the 6-month model and 0.73 for the 12-month model, indicating an acceptable/moderate goodness-of-fit level among both models^56^. The congruence of results and their interpretation amongst stepwise forward and backward selection models and those involving sensitivity analyses of the influence of supplements, contraceptives and/or medications suggested that for simplicity, it was appropriate to report the results for the overall sample.

**Table 2:** Logistic regression analysis of associations between PRMD onset and factors reflecting demographics, health-related status and the playing of musical instrument.

Variables	PRMD onset within 6 months		PRMD onset within 12 months	
	Coefficient (SE)	95% CI	Coefficient (SE)	95% CI
**MSK complaint at baseline** (vs noMSK at baseline)	0.415** (0.139)	(0.214;0.801)	0.441* (0.154)	(0.223;0.873)
**Δ BMI**	–		0.663* (0.113)	(0.475;0.928)
**Δ Physical activity participation levels** [IPAQ-SF score] (change vs no change)	2.343** (0.729)	(1.274;4.310)	2.346* (0.805)	(1.198;4.596)
**Perfectionism** [HFMPS-SF Δ SPP sub-scale score]	1.102** (0.040)	(1.027;1.183)	–	
**Δ Fatigue** [CFQ 11 score]	1.084** (0.027)	(1.033;1.138)	1.081* (0.012)	(1.018;1.148)
Constant	0.193*** (0.053)	(0.113;0.330)	0.765 (0.212)	(0.444;1.317)
Number of observations (n)	258	–	179	–
McFadden Pseudo-R^2^	0.131	–	0.126	–
Area under the ROC curve	0.722	–	0.728	–

(Standard errors in parentheses).

***p < 0.001, **p < 0.01, *p < 0.05.

Δ, evolution; BMI, Body Mass Index; CFQ 11, Chalder Fatigue Scale; HFMPS-SF, Hewitt and Flett’s Multidimensional Perfectionism Scale-short form; IPAQ-SF, International Physical Activity Questionnaire-short form; MSK, Musculoskeletal; PRMD, Playing-related musculoskeletal disorder; SPP, Socially-prescribed perfectionism.

In the 6-month model, the variables significantly associated with PRMD onset were self-reporting a MSK complaint at baseline (*AOR* = 0.415; *p* < 0.01), a change in the physical activity level (IPAQ-SF score) (*AOR* = 2.343; *p* < 0.01), higher rates of SPP sub-score (*AOR* = 1.102; *p* < 0.01) and fatigue (*AOR* = 1.084; *p* < 0.01). The 12-month model confirmed the associations between the baseline MSK complaint (*AOR* = 0.441; *p* < 0.05), the change in the physical activity level (*AOR* = 2.346; *p* < 0.05) and an increase rate of fatigue (*AOR* = 1.081; *p* < 0.05) with PRMD onset, but not the association between the latter and an increased SPP sub-score. Moreover, the 12-month model showed that an increase of one additional unit of BMI appeared associated with a decrease of 33.7% (i.e., 1–0.663 = 0.337) in the odds of developing a PRMD (*p* < 0.05).

## Discussion

This study focused on identifying the factors associated with increased risk of PRMD onset within a 12-month period among a study population of music students at different educational stages (i.e., from secondary to tertiary education). The present study offers novelty amongst a longitudinal design and data from a population of music students engaged in education within a wide range of universities in Europe.

The onset of PRMDs occurred in 49% of music students within 12 months. This is similar to the figure of 59% reported by Ballenberger et al.^34^ for the 33 music students free of baseline musculoskeletal health complaints (MHC) enrolled in their prospective longitudinal analysis. Although Ballenberger et al.’s study involved a smaller sample of 33 music students from a music university in Germany and 30 non-music controls, it is the only study that can be compared directly with the present study due to the paucity of other prospective longitudinal studies in the literature^31–33^. It was noteworthy that the current paper’s longitudinal findings conflicted in part with those from the RISMUS cross-sectional baseline study, which had previously revealed a significant relation between PRMD prevalence and being a first- or a second-year Masters student^14^. Nevertheless, such discrepancies may have been influenced by loss-to-follow-up and diminishing sample size amongst the longitudinal assessments of the current study. Nevertheless, the loss-to-follow-up and diminishing sample size amongst the follow-ups should be considered within the interpretation of these results.

Clarity in the distinction between PRMDs and non-PRMDs described previously^14^ assumed even greater importance amongst the longitudinal data and the potential for a new interpretation of antecedent factors in the onset of PRMDs. For example, Baadjou et al.^11^ found a low association between pain and PRMDs in a study among 130 university-music students in the Netherlands, suggesting that these two variables might be reflecting different concepts. The authors concluded that the results from studies considering pain and PRMDs cannot necessarily be combined because musicians do not always experience PRMDs as pain^11,57^. It is possible that PRMDs as defined by Zaza et al.^7^, might reflect an important component relating to function and disability (i.e., interference with playing ability) rather than to pain per se, even though the latter could still be a symptom.

Therefore, for the initial cohort of students without PRMDs (n = 442), students with generic MSK complaints have been included because it was assumed that they could represent an important risk factor for the onset of PRMDs^34,58^. Accordingly, the paper’s design offered specific consideration of MSK conditions and symptoms that affect the playing of music, allowing the identification of the factors associated with the onset of PRMDs. Interestingly, in the 6-month logistic regression analysis, the AOR of 0.415 for the baseline MSK complaints indicates that, keeping all the other variables at a fixed value, students with a MSK complaint at baseline assessment have a 58.5% (1–0.415 = 0.585) lower likelihood to have a PRMD onset (*p* < 0.01; see Table 2). Thus, the probability of developing a PRMD increases in music students who have not yet developed symptoms. Paradoxically, self-reporting MSK symptoms was found to be potentially protective for the onset of a complaint interfering with their playing ability (i.e., PRMD). Some students with MSK complaints at baseline may be speculated to have found effective strategies (e.g., change of playing technique or behaviours toward health) to accommodate the effects of such complaints on their ability to play their instrument optimally. Similarly, it could be hypothesised that students without a necessity for coping strategies because of the previous absence of a disorder might be provoked towards maladaptive behaviours as a disorder develops. Future studies might usefully assess the efficacy of early-intervention strategies involving attitude and habit changes on pre-PRMD practising behaviours and on the cascade towards the onset of PRMD symptoms. In addition, the findings of the current study could provide a starting point for a more in-depth consideration on Zaza et al.’s definition of PRMDs, while exploring characteristics specifically related to PRMDs in comparison to generic MSK complaints.

Interestingly, a change (i.e., either increase or reduction) in the level of physical activity was associated with the onset of PRMDs (*AOR* > 1; *p* < 0.05; see Table 2). This finding was inconsistent with earlier cross-sectional studies, which found no associations between physical activity and the presence of MSK complaints^6,13,19,59–64^. Nevertheless, since results were related to a change in the physical activity participatory level, rather than to an increase or decrease in physical activity intensity, they should be interpreted cautiously. For example, this could be interpreted as an adaptive behaviour in which musicians decrease their physical activity due to the fear of worsening their health conditions or because their symptoms hinder its execution. Since musicians already perform an activity with their body by playing the instrument, too high and potentially incorrect physical activity (e.g., with wrong intensity) may lead to overload. Future prospective studies with adequate experimental design sensitivities will be able to provide new results that might help to understand the extent of the association and the actual cause-effect relationship between these two variables.

Moreover, the findings showed that PRMD onset was significantly associated with higher increase in the socially-prescribed perfectionism (SPP) score in the 6-month logistic regression analysis (*AOR* > 1; see Table 2). According to Hewitt and Flett^49,65^, socially-prescribed perfectionism is focused on social expectations and demands, which are somewhat external (e.g., parental expectations). Specifically, this type of perfectionism represents the perception (veridical or not) that other people expect perfection from the individual^66^. Perfectionistic inclinations are characteristics of elite performers that often increase during the progress to higher levels of performance, as happens among athletes^67^. Given the nature of their activity, which is focused on the public or school expectations depending whether they are professionals or students, the association between PRMD onset with perfectionism suggests that the latter could influence the onset of physical symptoms. Unfortunately, current research is scarce concerning the association between PRMDs and perfectionism. The evidence on perfectionism resides only within a restricted number of studies that have considered perfectionism levels and from which conflicting results have emerged^68,69^. Amongst these competitive environments, music students should therefore acquire effective strategies to cope with the high expectations (especially from others) that might result in some sort of maladaptive perfectionism^70^, degenerating into disruptive physical symptoms and leading to increased levels of psychological distress^68^.

Nevertheless, this study’s longitudinal findings did not show any association between psychological distress and the development of PRMDs. The latter could be attributed to the choice of the questionnaire that was not specific for musicians and therefore might not have been suitable for this population. In relation to this, it would be interesting to develop an in-depth investigation or a Delphi study on the most appropriate and standard outcome measures to be used with musicians. Finally, it could be the case that music students are not truly affected by psychological distress, consistently with findings of a previous systematic review, which showed that trait anxiety was found not to be associated with complaints in multivariate analysis^24^. In order to confirm or disprove this result, further longitudinal studies, possibly including the disentanglement between PRMDs and generic MSK complaints, in accordance with previous recommendations from the performing arts medicine field^32,33^, are needed.

Importantly, the novel findings from the longitudinal study with its more robust experimental design characteristics, endorse those reported previously at the study’s baseline^14^, in which PRMD onset was significantly correlated with an increase in the levels of fatigue (i.e., *AOR* > 1 in the CFQ 11 score; *p* < 0.05; see Table 2). As fatigue might result from physical, cognitive and emotional exertion^71,72^, it might be assumed that many musicians experience MSK symptoms attributable to a biomechanical dimension, possibly due to overuse of tissues involved in the act of playing and therefore related to their musical activities through nociceptive (e.g., repeated and constant load) or neuropathic (e.g., repetitive mechanical load resulting in damage to the peripheral nerve) mechanisms^3^. Biomechanical approaches to the assessment of movement tasks and fatigue might usefully be combined with appropriate patterns of MSK complaints to quantify the strain generated in specific tissues during playing activity. With this possibility in mind, future research should also focus on effective approaches to identify successful strategies to address painful conditions by developing risk models that might be used to build safe and effective training guidelines for musicians^73^. A proper strategy for PRMD prevention and fatigue management could be a potential contribution within a healthier educational context and might reduce the impact of PRMDs among music students aspiring to become professional musicians^74^. Indeed, based on self-reported PRMD rates from the current literature, it seems that the prevalence of MSK complaints amongst professionals and music students is relatively unchanged over recent decades^33^. This is despite the prevention strategies that had been established in Europe^75–80^ and the UK^81,82^. It could be speculated that PRMD rates might be related to insufficient health promotion and inefficient prevention awareness during music students’ training. This indicates that better results could be obtained by addressing health awareness taking into consideration the findings of the present study and the latest research findings from prospective longitudinal studies to make them effective and functional resources for music students’ training. In order to optimise the effectiveness of prevention and clinical approaches, a close collaboration between music institutions and healthcare professionals is fundamental. Especially in primary and secondary prevention, proactive behaviours, such as providing music students with information about their condition and facilitating management options^83^, are key prerogatives for an effective preventive and clinical practice.

### Limitations

There are limitations to be aware of when considering the study’s design. As outlined previously^36^, the invitation for participants to complete the questionnaire was sent by the school registries, without the possibility of reinforcing the invitation by sending a reminder in another form (e.g., via a telephone interview). Reminder e-mails were sent to the school registries, along with flyers to be distributed at the conservatories, in order to persuade students participating in the research to complete the questionnaires at the baseline and at the follow-ups. Despite extensive attempts to keep students actively engaged in the study, some students dropped out during the first and second follow-ups. Hence, missing data might have seriously compromised the interpretation and the meaning of results. Indeed, there were only 118 participants included within the second follow-up and this reduction might have decreased the study’s experimental design sensitivity amongst a relatively large number of candidate variables (i.e., 21) contributing potentially to the onset of PRMDs. Nevertheless, the study’s principal aim was best served by a longitudinal design involving logistic regression analyses of data from the largest possible sample size at baseline (n = 442). Within this context and a time-limited follow-up of one year, factors could be considered only as being associated with the onset of PRMDs rather than having causal underpinnings. Despite this limitation for the interpretation of the findings, the study’s longitudinal approach could be considered as contributing meaningfully to the literature.

In relation to the concept of PRMD, its definition developed by Zaza et al.^7^ has the important advantage of excluding symptoms that do not have an impact or a negative effect on musicians’ playing activity^84^. Indeed, this definition has been adopted in the present study to focus on musicians affected by disorders that might elicit a significant limitation in function and a meaningful impact on their playing activity (i.e., interference with playing ability) rather than only focussing on symptoms. Nevertheless, as an independent variable, this operational outcome definition has the limitation of not being able to address all MSK health-related aspects and of not providing a causality of the disorder (i.e., the disorder is the result of playing the instrument). Additionally, this study might have benefitted from incorporating alternative, well-known and recognised definitions from established and leading sources, such as the International Association for the Study of Pain (IASP). As such, using the definition of Zaza et al. might have introduced a potential participant bias by the inclusion of only musicians with playing ability affected by PRMDs. However, although another gold standard definition for MSK complaints related to musicians’ playing activity does not currently exist^84^, it could be considered the best definition currently available^32^ that might help in the interrogation of the literature, by offering comparability with data from studies already published in this area. The last point was particularly important because the extensive heterogeneity of types of methods and definitions already evident amongst the literature, has made synthesis of the evidence limited, if not impossible^31,84^.

Another consideration was that the study couldn't access important data from those who didn't respond or left the study because there was no direct interaction with students. This data could have been valuable in evaluating any potential biases in the research results. For example, the authors couldn't rule out the possibility of a sampling bias. Furthermore, details about the student enrolment and participation in the study were unavailable to the authors due to the confidentiality of the data, and the authors had lacked formal permission to publish it. Nevertheless, despite the limitations and the exploratory nature of the data, the present study provided an original contribution for the interpretation of PRMDs and their associated burden among music students of different nationalities and at different levels of training.

## Conclusions

This longitudinal study revealed that the factors significantly associated with the onset of PRMDs during an assessment period of up to 12 months were changes in physical activity (both increase and decrease), increased levels of fatigue and an increased level of socially-prescribed perfectionism. The presence of MSK complaints at baseline and changes to BMI were significantly associated with limiting the onset of PRMDs among music students participating in this longitudinal study. The findings of the present longitudinal study might have implications for further research and clinical practice as preventive interventions and targeted treatment strategies for PRMDs derived from its evidence might offer enhanced effectiveness.

### Supplementary Information

Supplementary Tables.

## Data Availability

Data cannot be distributed publicly because they encompass potentially identifying or sensitive participants’ information. Additionally, disclosure to third parties has been forbidden by the QMU Ethics Committee. Data are only available for researchers who meet the criteria for access to confidential data and are stored at a secure server hosted by Queen Margaret University. These data can be made available to interested researchers upon request to the corresponding author, who will have to ask the permission for data access to the QMU Ethics Committee at ResearchEthics@qmu.ac.uk.
