# Supplementary material for: Factors associated with increased risk of playing-related disorders among classical music students within the Risk of Music Students (RISMUS) longitudinal study
Source: Sci Rep. 2023 Dec 22;13:22939. doi: 10.1038/s41598-023-49965-7 (PMC10746703; doi:10.1038/s41598-023-49965-7)
Supplement: Supplementary file 1 — Supplementary Tables. [file 41598_2023_49965_MOESM1_ESM.docx]

**SUPPLEMENTARY TABLE S1. Descriptive statistics at 6 and 12 months.**

| **Variable** | | **6-month analysis** | | **12-month analysis** | |
| --- | --- | --- | --- | --- | --- |
|  |  | **n** | **%** | **n** | **%** |
| **Gender** | Women | 164 | 60.5% | 114 | 58.2% |
| (n=271 6 months) | Men | 106 | 39.1% | 81 | 41.3% |
| (n=196 12 months) | Other | 1 | 0.4% | 1 | 0.5% |
|  |  |  |  |  |  |
| **Age in years**  (n=271 6 months) | median (IQR) | 22.0 | (5.0) | 22.0 | (5.0) |
|  |  |  |  |  |  |
| (n=196 12 months) |  |  |  |  |  |
|  |  |  |  |  |  |
| **Nationality (region)***  (n=271 6 months)  (n=196 6 months) | South Europe | 146 | 53.9% | 108 | 55.1% |
|  | West Europe | 90 | 33.2% | 68 | 34.7% |
|  | Other | 35 | 12.9% | 20 | 10.2% |
|  |  |  |  |  |  |
| **Academic level**  (n=271 6 months) | Secondary education | 25 | 9.2% | 22 | 11.2% |
|  | Bachelor 1&2 | 50 | 18.4% | 30 | 15.3% |
| (n=196 12 months) | Bachelor 3&4 | 53 | 19.6% | 42 | 21.4% |
|  | Master 1&2 | 20 | 7.4% | 14 | 7.1% |
|  | Master 3&4 | 80 | 29.5% | 54 | 27.6% |
|  | Gap year/continuing education | 43 | 15.9% | 34 | 17.4% |
|  |  |  |  |  |  |
| **BMI in kg/m^2^** | median (IQR) | 21.2 | (4.0) | 21.2 | (4.0) |
| (n=267 6 months) |  |  |  |  |  |
| (n=194 12 months) |  |  |  |  |  |
|  |  |  |  |  |  |
| **Self-rated health [SRH]** | Excellent | 27 | 10.0% | 21 | 10.7% |
| (n=271 6 months) | Very good | 93 | 34.3% | 64 | 32.7% |
| (n=196 12 months) | Good | 111 | 41.0% | 88 | 44.9% |
|  | Fair | 38 | 14.0% | 21 | 10.7% |
|  | Poor | 2 | 0.7% | 2 | 1.0% |
|  |  |  |  |  |  |
| **Hours of sleep** | median (IQR) | 7.0 | (1.0) | 7.2 | (1.0) |
| (n=271 6 months) |  |  |  |  |  |
| (n=196 12 months) |  |  |  |  |  |
|  |  |  |  |  |  |
| **Smoking** | Yes | 41 | 15.2% | 30 | 15.3% |
| (n=270 6 months) | No | 229 | 84.8% | 166 | 84.7% |
| (n=196 12 months) |  |  |  |  |  |
|  |  |  |  |  |  |
| **Medications** | Nothing | 228 | 84.1% | 167 | 85.2% |
| (n=271 6 months) | Supplement/ contraceptive | 23 | 8.5% | 14 | 7.1% |
| (n=196 12 months) | Medicine | 20 | 7.4% | 15 | 7.7% |
|  |  |  |  |  |  |
| **Physical activity participation levels**  **[IPAQ-SF score]** | High | 49 | 18.2% | 33 | 16.9% |
| (n=268 6 months) | Moderate | 130 | 48.3% | 98 | 50.3% |
| (n=194 12 months) | Low | 90 | 33.5% | 64 | 32.8% |
|  |  |  |  |  |  |
| **Psychological distress [K10 score]** | median (IQR) | 18.0 | (9.5) | 18.5 | (10.0) |
| (n=269 6 months) |  |  |  |  |  |
| (n=195 12 months) |  |  |  |  |  |
|  |  |  |  |  |  |
| **Perfectionism [HFMPS-SF score]** |  |  |  |  |  |
| **SOP sub-scale score** | median (IQR) | 25.0 | (10.0) | 24.0 | (10.0) |
| (n=265 6 months) |  |  |  |  |  |
| (n=192 12 months) |  |  |  |  |  |
|  |  |  |  |  |  |
| **OOP sub-scale score** | median (IQR) | 18.0 | (9.0) | 17.5 | (7.0) |
| (n=267 6 months) |  |  |  |  |  |
| (n=194 12 months) |  |  |  |  |  |
|  |  |  |  |  |  |
| **SPP sub-scale score** | median (IQR) | 17.0 | (7.0) | 17.0 | (7.0) |
| (n=268 6 months) |  |  |  |  |  |
| (n=195 12 months) |  |  |  |  |  |
|  |  |  |  |  |  |
| **Fatigue [CFQ 11 score]** | median (IQR) | 12.0 | (6.0) | 12.0 | (6.0) |
| (n=265 6 months) |  |  |  |  |  |
| (n=193 12 months) |  |  |  |  |  |
|  |  |  |  |  |  |
| **Instrument** [classification]** | Elevated both frontal | 15 | 5.5% | 13 | 6.6% |
| (n=271 6 months) | Elevated both left | 46 | 17.0% | 29 | 14.8% |
| (n=196 12 months) | Elevated left | 19 | 7.0% | 10 | 5.1% |
|  | Elevated right | 37 | 13.7% | 28 | 14.3% |
|  | Neutral | 116 | 42.8% | 90 | 45.9% |
|  | Voice | 38 | 14.0% | 26 | 13.3% |
|  |  |  |  |  |  |
| **Years of practice** | median (IQR) | 13.0 | (5.0) | 13.0 | (5.0) |
| (n=271 6 months) |  |  |  |  |  |
| (n=196 12 months) |  |  |  |  |  |
|  |  |  |  |  |  |
| **Hours of practice per day** | median (IQR) | 3.0 | (1.0) | 3.0 | (1.0) |
| (n=271 6 months) |  |  |  |  |  |
| (n=196 12 months) |  |  |  |  |  |
|  |  |  |  |  |  |
| **Perceived exertion after 45 minutes**  **of practice without breaks** | median (IQR) | 3.6 | (4.0) | 4.0 | (4.2) |
| (n=271 6 months) |  |  |  |  |  |
| (n=196 12 months) |  |  |  |  |  |
|  |  |  |  |  |  |
| **Preparatory exercises** | Yes | 118 | 43.5% | 90 | 45.9% |
| (n=271 6 months) | No | 153 | 56.5% | 106 | 54.1% |
| (n=196 12 months) |  |  |  |  |  |
|  |  |  |  |  |  |
| **Breaks during practice** | Yes | 173 | 63.8% | 127 | 64.8% |
| (n=271 6 months) | No | 98 | 36.2% | 69 | 35.2% |
| (n=196 12 months) |  |  |  |  |  |

BMI, Body Mass Index; CFQ 11, Chalder Fatigue Scale; HFMPS-SF, Hewitt and Flett’s Multidimensional Perfectionism Scale – short form; IPAQ-SF, International Physical Activity Questionnaire – short form; K10, Kessler Psychological Distress Scale; OOP, Other-oriented perfectionism; SOP, Self-oriented perfectionism; SPP, Socially-prescribed perfectionism.

*This classification was made according to United Nations, S. D. Standard Country or Area Codes for Statistical Use, Series M, No. 49 (M49) <<https://unstats.un.org/unsd/methodology/m49/>> (1999).

** The question at baseline and at each follow-up was to indicate their main instrument.

BMI, Body Mass Index; CFQ 11, Chalder Fatigue Scale; HFMPS-SF, Hewitt and Flett’s Multidimensional Perfectionism Scale – short form; IPAQ-SF, International Physical Activity Questionnaire – short form; K10, Kessler Psychological Distress Scale; OOP, Other-oriented perfectionism; SOP, Self-oriented perfectionism; SPP, Socially-prescribed perfectionism.

*This classification was made according to United Nations, S. D. Standard Country or Area Codes for Statistical Use, Series M, No. 49 (M49) <<https://unstats.un.org/unsd/methodology/m49/>> (1999).

** The question at baseline and at each follow-up was to indicate their main instrument.

**SUPPLEMENTARY TABLE S2. Descriptive statistics of the evolution of variables.**

| **Variable** | | **6-month analysis** | | **12-month analysis** | |
| --- | --- | --- | --- | --- | --- |
|  |  | **n** | **%** | **n** | **%** |
| **Δ BMI in kg/m^2^** | median (IQR) | 0.0 | (0.8) | 0.0 | (1.0) |
| (n=249 6 months) |  |  |  |  |  |
| (n=184 12 months) |  |  |  |  |  |
|  |  |  |  |  |  |
| **Self-rated health [SRH]**  (n=271 6 months)  (n=196 12 months) | Unchanged | 148 | 54.6% | 99 | 50.5% |
|  | Amelioration | 44 | 16.2% | 30 | 15.3% |
|  | Worsening | 79 | 29.1% | 67 | 34.2% |
|  |  |  |  |  |  |
| **Δ Hours of sleep** | median (IQR) | 0.0 | (0.6) | 0.0 | (1.0) |
| (n=270 6 months) |  |  |  |  |  |
| (n=194 12 months) |  |  |  |  |  |
|  |  |  |  |  |  |
| **Smoking** | Unchanged | 256 | 95.2% | 184 | 94.4% |
| (n=269 6 months) | Started | 8 | 3.0% | 8 | 4.1% |
| (n=195 12 months) | Quit | 5 | 1.8% | 3 | 1.5% |
|  |  |  |  |  |  |
| **Medications** | Unchanged | 260 | 95.9% | 189 | 96.4% |
| (n=271 6 months)  (n=196 12 months) | Quit medication | 10 | 3.7% | 7 | 3.6% |
|  | Started medication | 1 | 0.4% | 0 | 0.0% |
|  |  |  |  |  |  |
| **Δ Physical activity participation levels [IPAQ-SF score]** | Unchanged | 141 | 52.4% | 93 | 47.7% |
| (n=269 6 months) | Increase | 113 | 42.0% | 93 | 47.7% |
| (n=195 12 months) | Reduction | 15 | 5.6% | 9 | 4.6% |
|  |  |  |  |  |  |
| **Δ Psychological distress [K10 score]** | median (IQR) | 1.0 | (5.0) | 2.0 | (6.0) |
| (n=263 6 months) |  |  |  |  |  |
| (n=191 12 months) |  |  |  |  |  |
|  |  |  |  |  |  |
| **Perfectionism [HFMPS-SF score]** |  |  |  |  |  |
| **Δ SOP sub-scale score** | median (IQR) | 1.0 | (5.0) | 2.0 | (4.0) |
| (n=258 6 months) |  |  |  |  |  |
| (n=186 12 months) |  |  |  |  |  |
|  |  |  |  |  |  |
| **Δ OOP sub-scale score** | median (IQR) | 2.0 | (5.0) | 3.0 | (5.0) |
| (n=260 6 months) |  |  |  |  |  |
| (n=188 12 months) |  |  |  |  |  |
|  |  |  |  |  |  |
| **Δ SPP sub-scale score** | median (IQR) | 2.0 | (5.0) | 3.0 | (6.0) |
| (n=261 6 months) |  |  |  |  |  |
| (n=189 12 months) |  |  |  |  |  |
|  |  |  |  |  |  |
| **Δ Fatigue [CFQ 11 score]** | median (IQR) | 3.0 | (9.0) | 4.0 | (9.5) |
| (n=260 6 months) |  |  |  |  |  |
| (n=189 12 months) |  |  |  |  |  |
|  |  |  |  |  |  |
| **Δ Hours of practice per day** | median (IQR) | -0.5 | (1.0) | 0.0 | (1.0) |
| (n=268 6 months) |  |  |  |  |  |
| (n=195 12 months) |  |  |  |  |  |
|  |  |  |  |  |  |
| **Δ Perceived exertion after 45 minutes of practice without breaks** | median (IQR) | 0.0 | (2.3) | 0.0 | (2.5) |
| (n=270 6 months) |  |  |  |  |  |
| (n=196 12 months) |  |  |  |  |  |
|  |  |  |  |  |  |
| **Preparatory exercises** | Unchanged | 212 | 78.8% | 151 | 77.8% |
| (n=269 6 months) | Introduced | 33 | 12.3% | 29 | 15.0% |
| (n=194 12 months) | Stopped | 24 | 8.9% | 14 | 7.2% |
|  |  |  |  |  |  |
| **Breaks during practice** | Unchanged | 204 | 75.3% | 146 | 74.5% |
| (n=271 6 months) | Introduced | 48 | 17.7% | 36 | 18.3% |
| (n=196 12 months) | Stopped | 19 | 7.0% | 14 | 7.1% |
|  |  |  |  |  |  |
| **Technique change^1^ [number of]** | 0 | 73 | 26.9% | 48 | 24.5% |
| (n=271 6 months) | 1 | 126 | 46.5% | 90 | 45.9% |
| (n=196 12 months) | 2 | 72 | 26.6% | 47 | 24.0% |
|  | 3 | 0 | 0.0% | 11 | 5.6% |
|  |  |  |  |  |  |
| **Professor change^1^ [number of]** | 0 | 142 | 52.4% | 90 | 45.9% |
| (n=271 6 months) | 1 | 119 | 43.9% | 94 | 48.0% |
| (n=196 12 months) | 2 | 10 | 3.7% | 12 | 6.1% |
|  |  |  |  |  |  |
| **Instrument change^1^ [number of]** | 0 | 234 | 86.6% | 165 | 84.2% |
| (n=270 6 months) | 1 | 35 | 13.0% | 30 | 15.3% |
| (n=196 12 months) | 2 | 1 | 0.4% | 1 | 0.5% |
|  |  |  |  |  |  |
| **Perceived cause attributed to the**  **instrument** | Yes | 60 | 76.9% | 71 | 74.0% |
| (n=78 6 months) | No | 18 | 23.1% | 25 | 26.0% |
| (n=96 12 months) |  |  |  |  |  |

Δ, evolution; BMI, Body Mass Index; CFQ 11, Chalder Fatigue Scale; HFMPS-SF, Hewitt and Flett’s Multidimensional Perfectionism Scale – short form; IPAQ-SF, International Physical Activity Questionnaire – short form; K10, Kessler Psychological Distress Scale; OOP, Other-oriented perfectionism; SOP, Self-oriented perfectionism; SPP, Socially-prescribed perfectionism; SRH, Self-rated health.

^1^ The number of changes have been measured by asking the students if they changed technique, professor and/or instrument during the 6 months prior to baseline, 6 months follow-up and 12 months follow-up evaluations.

Elevated both frontal: Music students playing musical instruments with both arms elevated in a frontal position (i.e., harp, trombone, and trumpet); Elevated both left: Music students playing musical instruments with both arms elevated in the left quadrant position (i.e., viola, violin); Elevated left: Music students playing musical instruments with only the left arm elevated (i.e., cello, double bass); Elevated right: Music students playing instruments with only the right arm elevated (i.e., flute, guitar); Neutral: Music students playing instruments in a neutral position, without the elevation of arms (i.e., accordion, bassoon, clarinet, euphonium/tuba; French horn, harpsicord, oboe, organ, percussion, piano, recorder, saxophone).

## TABLE S3. Bivariate analysis. Significant associations between PRMD onset and factors reflecting demographics, health-related status and the playing of musical instrument.

| **Variable** | | **PRMD onset** | | | | | |
| --- | --- | --- | --- | --- | --- | --- | --- |
|  |  | **6-month analysis** | | **Statistical**  **test result** | **12-month analysis** | | **Statistical**  **test result** |
|  |  | **No** | **Yes** |  | **No** | **Yes** |  |
| **Nationality (region)** | |  |  |  |  |  |  |
| South Europe | | 77% | 23% | χ^2^(df, 2)=6.79* | - | | |
| West Europe | | 61% | 39% |  |  |  |  |
| Other | | 74% | 26% |  |  |  |  |
|  | | | |  |  |  |  |
| **Baseline MSK complaint** | |  |  |  |  |  |  |
| No MSK complaint | | 66% | 34% | χ^2^(df, 1)=7.40** | 44% | 56% | χ^2^(df, 1)=6.59* |
| MSK complaint | | 81% | 19% |  | 63% | 37% |  |
|  | |  |  |  |  |  |  |
| **Δ BMI** | |  |  |  |  |  |  |
| median (IQR) | | 0.0 (1.0) | 0.0 (0.5) | z=2.11* | 0.2 (1.3) | 0.0 (1.0) | z=2.88** |
|  | |  |  |  |  |  |  |
| **Δ Physical activity**  **[IPAQ-SF score]** | |  |  |  |  |  |  |
| Unchanged | | 80% | 20% | χ^2^(df,2)=12.03** | 60% | 40% | χ^2^(df, 2)=7.92* |
| Increase | | 61% | 39% |  | 44% | 56% |  |
| Reduction | | 60% | 40% |  | 22% | 78% |  |
|  | |  |  |  |  |  |  |
| **Perfectionism [HFMPS-SF]** | |  |  |  |  |  |  |
| **Baseline SOP sub-scale score** | |  |  |  | - | | |
| median (IQR) | | 26.0 (10.0) | 22.5 (11.3) | z=2.38* |  |  |  |
|  | |  |  |  |  |  |  |
| **Δ OOP sub-scale score** | |  |  |  |  |  |  |
| median (IQR) | | 2.0 (5.0) | 3.0 (6.0) | z=-2.49* |  |  |  |
|  | |  |  |  |  |  |  |
| **Δ SPP sub-scale score** | |  |  |  |  |  |  |
| median (IQR) | | 2.0 (6.0) | 4.0 (6.0) | z=-3.12** |  |  |  |
|  | |  |  |  |  |  |  |
| **Δ Fatigue [CFQ 11 score]** | |  |  |  |  |  |  |
| median (IQR) | | 1.5 (7.0) | 6.0 (10.0) | z=-3.84*** | 3.0 (8.0) | 6.0 (10.0) | z=-2.71** |
|  |  |  |  |  |  |  |  |
| **Δ Hours of practice per day** | |  |  |  |  |  |  |
| median (IQR) | | -1.0 (1.0) | 0.0 (1.0) | z=-1.97* | - | | |
|  | |  |  |  |  | | |
| **Δ Hours of sleep** | |  |  |  |  |  |  |
| median (IQR) | | - | | | 0.0 (1.5) | 0.0 (0.5) | z=2.18* |

*** *p* < 0.001, ** *p* < 0.01, * *p* < 0.05

Results of bivariate analysis derived from the overall sample and from a sub-sample of participants not taking any supplements, contraceptives and/or medications, did not reveal any significant variations or differences. This similarity amongst the findings indicated that the latter factors within the sensitivity analysis had not intruded substantively and accordingly, the overall sample’s results have been reported for simplicity. For categorical variables, the table reports the onset of PRMDs’ distributions (row percentages) for every category of the variable considered, as well as the chi-square statistic and its statistical significance level. For continuous variables, the table reports the median and the IQR for the two PRMD onset categories, as well as the *z* statistics and its statistical significance level.

Δ, evolution; BMI, Body Mass Index; CFQ 11, Chalder Fatigue Scale; HFMPS-SF, Hewitt and Flett’s Multidimensional Perfectionism Scale–short form; IPAQ-SF, International Physical Activity Questionnaire – short form; MSK, Musculoskeletal; OOP, Other-oriented perfectionism; PRMD, Playing-related musculoskeletal disorder; SOP, Self-oriented perfectionism; SPP, Socially-prescribed perfectionism.
